# Practicing Mindfulness through mHealth Applications: Emerging Adults’ Health-Enhancing and Inhibiting Experiences

**DOI:** 10.3390/ijerph19052619

**Published:** 2022-02-24

**Authors:** Greenberry Taylor, Carma L. Bylund, Amanda Kastrinos, Jordan M. Alpert, Ana Puig, Joanna M. T. Krajewski, Bhakti Sharma, Carla L. Fisher

**Affiliations:** 1 Department of Communication, Flagler College, St. Augustine, FL 32084, USA; jkrajewski@flagler.edu; 2 College of Medicine, University of Florida, Gainesville, FL 32603, USA; carma.bylund@ufl.edu; 3 Department of Psychiatry and Behavioral Sciences, Memorial Sloan Kettering Cancer Center, New York, NY 10022, USA; kastria@mskcc.org; 4 College of Journalism and Communications, University of Florida, Gainesville, FL 32611, USA; jordan.alpert@ufl.edu (J.M.A.); bhakti.bhakti@ufl.edu (B.S.); carlalfisher@ufl.edu (C.L.F.); 5 College of Education, University of Florida, Gainesville, FL 32611, USA; anapuig@coe.ufl.edu

**Keywords:** mHealth, mindfulness, emerging adults, mobile applications, adverse effects, health communication

## Abstract

Mindfulness-based interventions (MBIs) and practices (MBPs) can promote better health outcomes. Although MBIs and MBPs were developed to be delivered in-person, mobile health (mHealth) tools such as apps have made these more accessible. Mindfulness apps (MAs) are popular among emerging adults (EAs) who have the highest ownership of smartphones and who are also at risk for distress. While adverse effects have been observed with MBIs/MBPs, this has not been examined when mindfulness is practiced using apps. We interviewed EAs (*n* = 22) to capture their motivations for using these apps and identified health-inhibiting and enhancing experiences. Data were thematically analyzed using the constant comparative method. Motivations for app use included accessibility, convenience, and stress/health management. EAs described health-enhancing outcomes (reduced distress, improved physical symptoms, increased focus) and health-inhibiting outcomes (worsened distress, performance uncertainty, dependency development, worsened physical health). They provided suggestions for improving apps (e.g., feedback option). These findings illustrate benefits and risks that EAs may encounter when practicing mindfulness using apps, which can inform the best practices for app design.

## 1. Introduction

The Buddhist concept of mindfulness was adopted by Western medical practitioners in the late 1970s to create mindfulness-based interventions (MBIs) [1]. Medical practitioners and academic scholars have operationalized mindfulness over time, with most definitions highlighting that it is a state of awareness that an individual experiences when purposefully paying attention to the present-moment nonjudgmentally as events unfold [2,3]. Meditation, yoga, and deep breathing are among the most well-known Buddhist mindfulness-based practices (MBPs) used to cultivate mindfulness [4]. Interventions such as John Kabat-Zinn’s mindfulness-based stress reduction (MBSR) [5] program were designed using these techniques (e.g., informal meditation, hatha yoga), and other well-known MBIs, including mindfulness-based cognitive therapy (MBCT) [6], incorporated elements of MBSR in addition to cognitive-based therapy techniques.

These interventions have been used in treatment plans for individuals with chronic and acute health conditions. Research has shown empirical evidence that MBIs (traditionally delivered face-to-face) have improved the quality of life and well-being of individuals with conditions such as arthritis and type 2 diabetes [7], fibromyalgia [8], cancer [9], schizophrenia [10], and anxiety and depression [11,12]. Findings from randomized-control trials (RCTs) using MBIs have shown that they are effective in improving mental health and reducing the risk of depressive relapse [13]. Additionally, standalone mindfulness practices have also been associated with positive health outcomes. For example, an international review of comparison studies using yoga found reported improvements in areas such as energy, sleep, and pain [14], while a survey of meditators in the US reported perceived improvements in social relationships, stress levels, and regulation of emotions [15].

With the emergence of new technologies, MBIs and MBPs no longer rely on face-to-face delivery and are widely available outside of clinical or therapeutic settings via mobile health (mHealth) tools; mHealth is defined as mobile devices such as smartphones and patient monitoring devices that support both medical and public health practices [16]. Among the more popular mHealth delivery systems of mindfulness, there are mobile applications (apps). In 2015, 65% of all healthcare app downloads were wellness management apps [17]. Calm, a popular mindfulness app (MAs), was the 20th overall ranked app on iOS (Apple Store) and top-grossing health and fitness app in 2019 [18]. During the COVID-19 pandemic, downloads for MAs saw an unprecedented rise, surpassing 750,000 downloads in just the last week of March 2020 [19]. Despite the continuous rise in mainstream adoptions of MAs, scholars have referred to apps as, “The new frontier of completely unsupervised meditation in mass quantities” [20] (p. 1).

Apps are easily accessible, particularly for emerging adults (EAs; ages 18–29), who account for the highest ownership of smartphones in the United States (96%) [21]. However, the idea of unsupervised practice of meditation is concerning, especially when considering these apps are available to at-risk populations and that health-inhibiting outcomes, including mental and somatic distress (e.g., increased anxiety, acute psychosis, somatic pain), have been linked to MBIs and MBPs [22,23]. EAs are particularly vulnerable to mental health distress because they are at a point in their lifespan when their brains are still developing, and they are encountering stressful life changes central to their identity development and independence [24]. Although traditionally delivered (in-person, face-to-face) MBIs and MBPs have reported mental and somatic distress in individuals [23], there is a dearth in literature focusing specifically on the emergence of health-inhibiting effects related to MAs. With this in mind, this study aims to explore potential health-enhancing and health-inhibiting outcomes of MAs experienced by an at-risk population: emerging adults.

### 1.1. Health-Inhibiting Effects of Mindfulness

Research documenting health-inhibiting outcomes of MBIs and MBPs, often referred to as adverse effects, is limited, despite “meditation casualties” (e.g., suicidal attempts, increased depression) being cited in practitioners of transcendental meditation (TM) in the 1970s [25]. Recently published narrative reviews [22] have cited instances where individuals engaging with MBIs and MBPs have experienced varying degrees of adverse effects, ranging from moderate (e.g., increased anxiety, depression) to severe (e.g., schizophrenic episodes, meditation-induced psychosis) [23]. These effects have been documented in both inexperienced practitioners, as well as Buddhist experts and practitioners [26]. Researchers examining health-inhibiting outcomes in this area often focus on individuals that have participated in an MBI or those that regularly engage with an MBP. In doing so, beginners or non-experts have been excluded in addition to those who participate in secular mindfulness.

Limited research examining users’ experiences with MAs has been conducted; however, some studies have identified health-inhibiting effects. Laurie and Blandford [27] examined nurses’ experiences with Headspace, an MA that provides guided and unguided meditation activities. Findings revealed that half of participants reported feelings of frustration or the inability to control their thoughts when entering a session with a stressful or agitated state-of-mind. Clarke and Draper [28] examined college students’ experiences with Calm’s “7 Days of Calm” program and noted that participants at times felt worse after using the app or that it created feelings of low self-efficacy. Despite the ubiquity of MAs, to the best of our knowledge, no studies to date have explored MA users’ health-enhancing and health-inhibiting outcomes. This includes the experiences of EAs who are not only more likely to use MAs, but also may be at an increased risk of health-inhibiting effects.

### 1.2. Emerging Adults and Mindfulness Apps: A Need for MA Best Practices

EAs are in a peak developmental period, in which they are transitioning from adolescence to adulthood, a period that is characterized with both instability and self-exploration [24,29]. As previously mentioned, this instability (e.g., searching for identity, being separated from family, etc.) can contribute to EAs being more vulnerable to mental health distress and the development of issues such as anxiety and depression [30,31]. For instance, recent data show that rates of anxiety and depression have steadily increased in college students over a 10-year period (from 2008–2018): 10.2% to 17.3% (depression) and 10.4% to 22.0% (anxiety) from 2008 to 2018 [32,33]. EAs are also at a point in their lifespan when existing mental health disorders, such as schizophrenia, peak [34].

With access to smartphones and the ability to download apps, EAs experiencing mental health issues might be drawn to MAs because apps such as Calm and Headspace frequently advertise the alleviation of symptoms of anxiety and depression. Although some studies have highlighted the health-enhancing benefits of MAs for individuals at this point in the lifespan, specifically college students [35,36], the combination of access and vulnerability could be problematic for EAs who engage with MAs without warnings about risks, especially without any clinical oversight or therapeutic guidance [20]. The convenience, affordability, and accessibility of MAs could make them a valuable tool to help EAs manage their mental health, but research must be conducted to understand how they are negatively affected by the apps and develop recommendations to promote safe usage.

To date, researchers have not carefully elucidated the potential effects (both health-enhancing and health-inhibiting) of MAs for EAs. Given that MAs typically do not provide a statement of the potential risks and health-inhibiting effects [37], the primary aim of this study is to investigate EAs’ comprehensive health experiences with these apps in order to help guide their use of MAs and generate the best practices for app developers that could promote healthier outcomes. We posited the following inquiries:

**RQ1** **:** *What motivates EAs’ use of MAs?*

**RQ2** **:** *What health-enhancing outcomes do EAs associate with using MAs?*

**RQ3** **:** *What health-inhibiting outcomes do EAs associate with using MAs?*

**RQ4** **:** *What improvements to design do EAs suggest to promote healthy outcomes with MAs?*

## 2. Materials and Methods

### 2.1. Design

We used an interpretive, exploratory approach to prioritize EAs’ voice in identifying their health-enhancing and health-inhibiting experiences to promote healthier outcomes in their use of MAs [38]. This approach empowers individuals to share their first-hand experiences using detailed examples [39] and promotes the researcher’s ability to understand lived experiences from a more naturalistic perspective [40].

### 2.2. Sampling and Recruitment

Upon Institutional Review Board approval from the University of Florida (IRB201903276), we used purposive sampling to recruit EA users of MAs via multiple channels, including targeted Facebook advertisements, a university study website (SONA), and ResearchMatch, a national database of registered volunteers willing to participate in research studies. We developed a 13-question online screening survey to assess participants eligibility, which was distributed on all channels. Participants were eligible if they were between the ages of 18–29 and experienced both health-enhancing and health-inhibiting outcomes in conjunction with using an MA. The screening survey yielded 552 responses. Sixty individuals were eligible, and all were invited to participate in a 45–60 min interview, with a $20 Amazon gift card offered as compensation. Of those invited, 23 EAs participated in interviews. All participants gave their informed consent for inclusion before they participated in the study.

### 2.3. Procedures

Individuals participated in an in-depth, semi-structured, audio-recorded interview. A modification of the critical incident technique (CIT) was used, in which participants were asked to recall and describe in detail specific critical health incidents and were provided with examples to probe their memory (e.g., “Think of a time when you used the mindfulness app and you experienced healthy outcomes or effects”; “Think of a time when you used the mindfulness app and you did not experience healthy outcomes and instead had a negative experience or it was health-inhibiting in some way.”). The CIT helps individuals recall specific incidents, eliciting richer or more detailed accounts [40,41,42,43]. This technique has been used in a variety of sensitive or vulnerable health contexts, including sexual health in cancer survivorship [44], dementia patients’ emotional disorders [45], and veterans’ mental health [46]. After the interview, participants were given a list of mental health resources to provide additional support, should speaking about their experiences elicit mental distress. Interviews were professionally transcribed, resulting in 200 pages of data.

### 2.4. Analysis

Data were thematically analyzed using the constant comparative method [47,48]. This included concurrent data collection and analysis to achieve thematic saturation. Open coding involved multiple steps as outlined by Corbin and Strauss [49]: (1) keeping memos during interviews of emergent patterns; (2) assigning codes (labels) to concepts; (3) constantly comparing participants’ responses; (4) collapsing codes into categories (i.e., themes); (5) axial coding to identify thematic properties; (6) keeping memos for thick, rich description and exemplar quotes [50]. Codebooks for each inquiry were developed by the lead author and revised across the analytical process. Thematic saturation was achieved using well-established criteria (frequency, strength, and repetition) [51] and occurred after the 14th interview. Eight additional participants already enrolled were interviewed to ensure rigor by confirming saturation had been met.

The first (G.T.) and senior author (C.L.F.), experts in qualitative methodology, oversaw the analysis and reviewed data for each theme and thematic property. Once the codebooks were finalized, the analyses were validated by an additional coder (B.S.) using 60% (*n* = 12) of transcripts. Multiple verification strategies were used to ensure rigor, including using purposive sampling, conducting data collection and analysis concurrently, assigning theme labels in vivo, conducting additional interviews to confirm saturation, member-checking to confirm interpretation of data, and having multiple coders [52,53]. Participants are identified using a numbered coding system in parentheses to maintain confidentiality in the presentation of findings. The names of specific apps are used when mentioned by participants, and the term “app” was used when unspecified.

## 3. Results

We conducted 23 in-depth interviews with EAs, with one interview being excluded from analysis due to the participants’ misunderstanding of the study’s purpose of identifying health-enhancing and inhibiting experiences using MAs. Instead, the participant discussed negative aspects of the app that were not health related such as apps’ cost, usability, and functionality. Demographic information of participants (*n* = 22) included in analysis can be found in Table 1.

Findings are reported for each research inquiry. All participants received the same question to address RQ1. Thus, responses were grouped into categories with frequencies reported to illustrate the driving factors motivating use of MAs. A majority cited easy access a motivation (*n* = 13), and only a limited number cited their mental health (*n* = 4). Participants’ motivations and reasons can be seen in Table 2.

For RQs 2–4, participants’ lived accounts were used to illustrate health-enhancing and health-inhibiting experiences with MAs. Themes are identified in italics, and thematic properties are identified in bold, to further characterize these health outcomes.

### 3.1. RQ2: Emerging Adults’ Perceptions of App-Related Health-Enhancing Outcomes

All participants described experiencing at least one health-enhancing outcome that they associated with using the MA. These effects covered the spectrum of health outcomes including mental, physical, and emotional health experiences. Participants described health-enhancing experiences in three ways: (1) it calms me down; (2) I physically feel better, and (3) I can focus.

#### 3.1.1. It Calms Me Down

Participants frequently described the app as contributing to a sense of calm or that “it really calms me down” (18). They described an enhanced psychological state in that the app (1) reduced mental health distress and (2) alleviated stress.

**Reduced mental health distress:** Participants shared that using the app reduced mental health distress related to diagnosed disorders (e.g., generalized anxiety disorder (GAD), obsessive-compulsive disorder (OCD)). In other instances, it helped them manage the psychological (e.g., feeling anxious or depressed) and physiological (e.g., panic or anxiety attacks, rapid heartbeat) symptoms of mental distress.

Participants linked their app usage to experiencing fewer symptoms of mental health distress or major episodes. One participant experienced “really bad depression/anxiety” that she believed should have resulted in her hospitalization. She explained that once she began meditating and using Headspace, the frequency of the episodes decreased, saying: “I genuinely feel like I experienced peace and happiness for the first time in my life. And in the past year, I’ve had one bad depressive episode … and there was a lot of shit going on [at that time]” (29). Similarly, another participant diagnosed with OCD noted that using the app helped to alleviate sensations he experiences during panic attacks. He recalled:

*When I’m studying or doing a practice exam I can freak out and have this crazy panic attack. I just pull out my phone, and I go on the app and do a three-or ten-minute meditation. I usually go to breathing exercises because it really calms me down. (18)*

At times, participants spoke about specific mental health disorders such as OCD, while others described how MAs helped them manage broad anxiety-related distress. For example, a Latina participant described feeling anxiety associated with attending a predominantly White college. She recalls her anxiety dissipated after she began using Headspace and meditating, stating:

*I couldn’t relate to anyone in my classes or anything. I’d get really anxious when I got home from not really knowing how to talk to people. So finally, when I started downloading different apps … it really helped me calm down after a long day. (22)*

A participant who experienced “anxious tics” shared how using MAs’ guided meditations alleviated mental distress, saying it helped ensure the anxiety “didn’t take over my whole body as it usually does” (25). Likewise, participants described feeling “more at peace” overall and that using the app helped with their moods.

**Alleviated stress:** Participants described the app as helping them manage their general feelings of stress or situational stress, as this participant shared: “[The app] helped me just to relieve that stress, to feel a little bit more relaxed” (19). Others spoke about how the app relieved stress specific to school or work, helping them cope and feel less overwhelmed by situational stressors such as deadlines, as this participant described:

*I definitely used [Calm] during midterms and finals when I had a lot going on. Sometimes I just need a mental break because of all the time that I’ve been studying. When I use the app it’s like it takes my mind away from all the schoolwork, responsibilities, and tasks I need to complete. (26)*

Another respondent, a high school teacher, explained how MAs have helped her in “high-stress situations,” when she is “having trouble bringing [her] own cortisol levels down.” She provided examples of these situations and how she approaches them since using MAs, saying:

*If we have a lockdown drill at school, or there’s a fight or something, I can feel my body reacting to it. I need to do something to help myself come down because the instinct is to just jump back into whatever you’re doing. But sometimes you need to be like, “Okay, I don’t feel my best. What can I do to kind of like bring myself down?” (17)*

#### 3.1.2. I Physically Feel Better

Participants described improvements to their physical health. This was interrelated to the previous theme, as participants described mental and physical health as interconnected (e.g., participants experiencing an anxiety or panic attack described an elevated heart rate). Physically feeling better was related to long-term physical changes, as this participant explained: “My physical health was also really bad. … I literally was known to be constantly sick, and I’m never sick now!” (29). Participants highlighted two improved aspects of physical health: (1) enhanced sleep and (2) improved physical ailments.

**Enhanced sleep:** Participants reported that MAs helped enhance their sleep in multiple ways, including falling asleep more easily, improving sleep quality, and feeling less tired during the day. Some participants expressed that the apps helped with previous sleep issues (e.g., insomnia), as this participant shared:

*I suffer from insomnia too, so [the app] lets me go to sleep instead of staying up all day, all night worrying about things. This definitely gives me a chance to rest instead of being half asleep for every damn day. (24)*

Others noted that they slept better in general, explaining that the app content guided them through the process of clearing their mind, which allowed them to fall asleep easier:

*I used [Headspace] when I was having a really hard time sleeping. I mainly use the sleep, and stress and anxiety [meditation content]. It would coach me through clearing my mind enough so that I could relax, and my body and mind can shut down for the night and go to sleep. (30)*

Another participant reported how this helped her sleep quality, saying “[Calm] does get me to a place where I can sleep faster and promote a better, deeper quality of sleep” (32).

**Improved physical ailments:** Participants also expressed that their physical ailments improved, including fewer migraine headaches, reduced symptoms of irritable bowel syndrome (IBS), and more energy, as described by this participant:

*I get bad migraines and IBS. It was like almost every day. I was nauseous and throwing up, or at least like once a week I would have a migraine. [Once I started using Headspace] I’ve gotten a migraine twice in the past year. I used to get sick all the time, too. I haven’t been sick at all. (29)*

Another participant said that before using apps she was “constantly tired throughout the day”, despite having slept well during the night and taking naps during the day. She explained that using Calm helped with her energy level, saying, “I found myself just overall feeling more energized throughout the day, and I didn’t feel as tired going about my day” (26).

#### 3.1.3. I Can Focus

Participants also reported improvement in their ability to focus, which was interrelated to feeling better physically (e.g., better sleep) and mentally (e.g., less overwhelmed/stressed). Although only three participants reported this effect, all three specifically mentioned an improved ability to focus and described it in terms of one specific health-enhancing effect: increased productivity.

**Increased productivity:** Participants described being more productive in areas such as everyday tasks and school. Increased productivity was also interconnected to other health benefits, such as getting better sleep. Participants illustrated how using Headspace cleared their mind to focus and be more productive, as this one shared:

*What’s helped me is that I feel like I can focus a little bit better. I feel like I’ve taken out the trash in my body. It feels like I’m less overwhelmed over things. And I’m not saying it’s always like this, but I think it does help in terms of my productivity and my attention. (28)*

### 3.2. RQ3: Emerging Adults’ Perceptions of App-Related Health-Inhibiting Outcomes

Almost all participants (with the exception of one) encountered at least one health-inhibiting effect they associated with using MAs. These experiences included both psychological and physical health outcomes. Health-inhibiting effects emerged in four ways: (1) sometimes it backfires; (2) I had performance uncertainty and anxiety; (3) you become dependent; (4) physically it was making it harder.

#### 3.2.1. Sometimes It Backfires

Some participants felt that using the apps had a negative impact on their mental health, either worsening their stress or creating additional stressors. These negative effects were associated with (1) worsened mental distress and (2) unwanted and intrusive thoughts.

**Worsened mental distress:** Participants described feeling worse during or after using the apps. At times, they described using apps to address existing issues (e.g., feeling anxious or stressed), but they said that it “backfired,” exacerbating their mental distress instead. This participant shared that using the app while “some trauma” was present intensified those feelings, saying: “It brings emotions, and it makes it feel really real. … Sometimes it backfires, and it just creates more anxiety for me” (24).

*At times, participants attributed an increase in distress to specific elements of the app they used. For instance, this participant using the “managing stress” program offered through Calm said, “I don’t find those to be effective for me. I feel like they naturally ramp up my anxieties, and they do the opposite of the health benefits on the account that they make me more anxious” (32). Another participant mentioned that the accessibility of MAs allowed him to meditate more often, which deepened feelings he was experiencing, saying: “I think the apps brought on more in the sense that they intensified. … I think the faster you start unearthing stuff like this, the faster it gets intense!” (11).*

In addition to amplifying existing feelings, participants described how using MAs sparked new negative feelings. This participant reported that the app put him in a state of being “either really sad or very depressed at that time” (24), while another said he experienced feelings of hopelessness after using Headspace. This participant explained why the app had this effect:

*I sometimes even felt worse because I’m actually paying attention to my body. I’m actually thinking about my stomach and feeling butterflies. And I think that when you’re actually feeling a little bit more severe symptoms it doesn’t feel great. (28)*

**Unwanted intrusive thoughts:** Participants reported experiencing unwanted intrusive thoughts after using the app. Several described how the app brought up painful memories, as this participant shared:

*I went on [Headspace] for a solid four days, and it brought back some memories that I have stored in the back of my head that I would never think about. And that’s when I was like, “All right, this is it. We’re not doing this on a normal basis” (14)*

Participants also mentioned feeling irritated or anxious during/after using apps. They said the feelings came from thinking that they should be working on other things and that meditating was a waste of time. For example, participants said:

*I stopped [using Headspace] for a while because it can make me really anxious because I do have a lot of thoughts in my mind. Like any other normal human, I have a lot going on. And so by turning off my brain, essentially, and just being there and existing it’s like, “Oh wait, by the way, you have like a paper due at the end of the week and you have to read these two textbooks and you have all of this other stuff.” And then it’s like, “Oh, why am I sitting here doing this when I have so much other stuff going on?” (13)*

*By the end by the end of the month [of using Calm], I was irritated that I had to use it for 10 min every day. During the meditation sessions, I would just be thinking about how I could be using that time to study or do homework instead. (12)*

#### 3.2.2. I Had Performance Uncertainty and Anxiety

Participants shared that using apps created uncertainty and anxiety related to their performance, saying: “[I] worried that I might not be doing it right or I’m not doing it the right way” (25). They described their performance uncertainty and associated anxiety in three ways: (1) encountered persistent uncertainty; (2) felt pressure to perform; and (3) wanted feedback.

**Encountered persistent uncertainty:** Participants reported becoming hyper-focused on their performance when using MAs, experiencing persistent thoughts of uncertainty and increasing feelings of anxiety and stress. For example, this participant recalled using Headspace to cultivate empathy/kindness but instead becoming focused on his performance, saying, “Maybe I’m not doing this [meditation activity] right? Maybe I have to switch to something different?” (19). Another participant explained how worry over her performance increased her anxiety:

*A lot of the times I would be on the brink of an anxiety attack and try to meditate to slow myself down. And it would just give me more content for furthering myself toward anxiety attacks. “Am I doing this correctly? Am I not meditating well? … Why can’t I focus directly on this app?” (27)*

Participants also mentioned feeling discouraged after sessions due to this uncertainty. This participant recalled that she spent mediation time on Headspace wondering if she was doing it correctly, which negatively impacted her experience. She said, “If you’re sitting there and you’re just like, ‘What’s wrong with me? Why does this relax everyone else, but for me, sometimes it’s just the worst five minutes of my life.’” (13).

**Felt pressured to perform.** Participants also disclosed that they felt pressured to use the app and relax, which negatively affected their experience, as this participant expressed:

*There’s this immense pressure to feel relaxed. And having to feel pressure to be relaxed, you’re not going to relax. … Sometimes the app can make it worse because they will send you like those notifications that say, “You haven’t practiced mindfulness in 10 days!” (13)*

A participant that experienced frustration and anxiety trying to follow the app’s instructions said:

*If I wasn’t sitting that way [meditation instructions], it would make me more anxious that they were saying that your hands had to be placed a certain way. Or there were certain things you had to do with your body when it was just a basic meditation of just sitting and doing breathing techniques. I think that one kind of frustrated me and made me more anxious. (25)*

**Wanted feedback:** Participants struggled with not receiving feedback and wanted to discuss their performance during/after the meditation. The absence of two-way communication was linked to both their feelings of persistent uncertainty and performance pressure, as this participant described:

*If I had some kind of question, I’d just be so stressed out because I’m like, “Oh my gosh, am I doing this correctly?” Like, it’s an app. I can’t just talk to the app and expect a response. I like having that reassurance. It was pretty stressful.” (18)*

Participants also noted that the lack of feedback impacted their ability to tailor their meditation/app activity to the particular sensations they were experiencing in the moment, as this participant explained, “By myself I don’t have the ability to do that [pivot to a different activity] because I’m not a trained psychologist to know how to properly use the app” (27). Another participant described how the lack of feedback and overall human connection on the app affected her experience:

*I think the thing is the app [Headspace] is very faceless. There’s not a person. There’s not my teacher who I’ve worked with for two-ish years now, who I have a good rapport with, Who I can be like, “You know, today’s session really didn’t work for me. Do you have any suggestions for me to try again later?” I can’t get feedback from the app, and I think that leads to the frustration. (21)*

#### 3.2.3. You Become Dependent

Participants described becoming dependent on MAs, feeling as though they had to rely on the app to manage certain health concerns. They described feeling dependent on the app for managing two health issues: (1) mental distress and (2) sleep.

**Mental distress:** Participants often reported feelings of frustration or disappointment after realizing that they relied heavily on the app. One explained:

*I feel like there was a certain point that I couldn’t keep my anxiety low unless I meditated. … It was frustrating that I couldn’t handle my own emotions and my own stress and anxiety because I was so dependent on the app. (22)*

Another participant described feeling anxious after losing access to certain Headspace features when his free trial expired:

*It was so difficult because sometimes I’d just wake up and go on my phone and go to the app. And I’m like, “Damn I just forgot the trial ended.”… I might sound crazy, but I was just so disappointed. And my anxiety was bad because I don’t have my videos anymore. I was just upset. (18)*

**Sleep.** Some participants reported becoming dependent on the app to achieve good sleep. One participant illustrated this saying:

*I feel like I did need it [Headspace] just because I needed to drown out my thoughts. … No one was going to sit next to me and talk to me while I try to fall asleep—that’s just unrealistic. So, I kind of did become dependent on it. (30)*

Another participant explained that they were not able to manage their sleep without assistance from the app, saying:

*I feel like the whole point of these apps is to better help manage your stress throughout your day, and help you sleep better throughout the night…on your own without needing to use the app…I didn’t feel like I was able to manage myself on my own. So, I had to use the app and technology in order to help myself. (26)*

#### 3.2.4. Physically It Was Making It Harder

While only five participants shared that using MAs also negatively impacted their physical health, they all explained that the app had caused them to experience some level of somatic distress.

**Somatic distress:** Participants described feeling disruptions in areas such as their sleep, appetite, and energy. One participant illustrated these disruptions, saying:

*I stopped using Headspace because it just got to a point where my body felt like it was asleep. … My body was completely relaxed, but my brain was still on. And once the meditation was over, I’d be like, “Okay, I’m awake still.” And I wasn’t really sure how to go back and turn it off again. (13)*

Another participant noted how the app impacted their energy level, saying:

*I have to catch myself sometimes. [The app] can make me feel lethargic ... I already have a slightly low blood pressure, so it might make me feel a little lightheaded if I do sessions that are too long. … But I feel like being relaxed is not the same as being lethargic. Because you can be relaxed and still be productive, but I was not being productive. I didn’t have the motivation to continue. (20)*

### 3.3. RQ4: Emerging Adults’ Suggestions for Improving Apps

More than half of the participants provided suggestions for improvements in MA design that could eliminate unhelpful or negative experiences they experienced. Emerging adults’ suggestions for improving apps included: (1) having additional resources and (2) having more tailored/customizable options.

#### 3.3.1. Having Additional Resources

Participants suggested adding resources and new features to improve their experience. These included features to (1) talk to a real human; (2) have a user check-in feature; (3) explain pros and cons; (4) enable community chats.

**Talk to a real human:** Participants proposed adding a feature that allows users to speak with someone in real-time via mobile-chat/phone/teleconference about their experiences on the app. This participant hoped that, with this feature, users could “actually talk to a real human that works for the app” that “gives them personal feedback” (14). Similarly, another participant said users “should be able to talk to a teacher or at least a licensed therapist” (11). A participant who described wanting feedback from the app suggested that having clinical “24/7 support” would be an innovative feature, explaining:

*It would be really unique and cool to have a psychologist connected through the app or having a support chat; something where if you have any questions about a specific video, or anything, you can just send a message and the person who responds is a doctor. (18)*

**User check-in feature:** Participants suggested a feature that inquires about users’ feelings (emotional and physical) when opening the app and before and after engaging in an activity. These responses could be used to recommend specific activities or connect users to outside resources. For example, this participant suggested that if a user reported having a negative experience with a certain activity, the app could give users advice on why this occurred and prompt them to “try something a little different” (21). Another participant who uses Calm and Breathe pointed out that these apps do inquire about feelings and moods, but they do not make recommendations for certain activities based on this information (24).

**Explain pros and cons:** Participants recommended adding information outlining potential health-benefits and health-inhibiting effects associated with mindfulness and emphasized the importance of making users aware of both. For example, this participant said that providing this information could have helped with her uncertainty and performance anxiety while using the app:

*I gave up because I thought that I was not doing it right or felt broken because I was struggling … But if you were greeted when you downloaded the app with, “Hey, it can benefit you in these ways, but you might also experience these [health-inhibiting effects], and that’s okay,” it would be normalizing the negative experiences. (13)*

Participants acknowledged that this information might already be in an apps’ Terms and Conditions; however, they also commented on the poor readability of these documents and how this information might be overlooked. They suggested using clear language and making the Terms and Conditions more accessible in the app, as this participant explained:

*[The app should] maybe provide something that says people might experience a certain level of adverse effects because I don’t think I have read that in the apps. Usually, these apps provide a very biased point of view that mindfulness is seen as something that is completely positive and forget to provide a disclaimer. (19)*

**Community chat:** Participants proposed having a forum where users could communicate with one another about their experiences, ask questions, and provide support. This participant suggested:

*[Have] a community chat function in the app where everyone who’s using the app have a chat feature and can be like, “Hey, does anyone experience this? I just did this and now I feel way more stressed out.”…People can communicate and build community. (29)*

Participants also described how a community forum could be another source for information about how to use the app, saying: “I know a lot of times people turn to other people that are using the app, and I think they find answers really quickly that way. So, something like that might be useful” (14).

#### 3.3.2. Have More Tailored/Customizable Options

Participants suggested that MAs should allow users to tailor content to better fit their specific goals and needs. Suggestions were presented as two ideas: (1) increased customization options and (2) usage limit.

**Increase customization options:** Participants who suggested the need for increased options spoke broadly about customization. This participant suggested making apps more “interactive” for users with short attention spans and offered an example of how this might be accomplished: “While you’re listening to the audio file, you’re also doing something in the app and focusing on that too” (31). Another suggested that apps could allow users to customize content specifically for them, stating: “Everyone’s vision of their mindfulness is different…I think it would be really cool if there was an app that can make it more familiar and customizable, but for your specific mindfulness techniques” (18).

**Usage limit:** The suggestion for a time limit was offered by some participants to decrease exposure to mindfulness techniques too fast, which could lower occurrences of health-inhibiting effects. They also felt that it would help prevent the development of dependency. For example, one participant that became dependent on the app said, “I wish it would have had a limit on how much you can use it for the first couple of times” (22). Another participant suggested that a time limit could be health promoting. He said:

*I think for the average person the health promoting thing would be to limit the usage to five minutes a day. Go the direction that workout apps go where the workout app promises you that just seven minutes a week you can get ripped. (11)*

## 4. Discussion

Emerging adults experienced both health-enhancing and health-inhibiting effects when using MAs. This research revealed that EAs engaged with these apps because of the ease of access and convenience they offer, and they perceived the apps as tools to potentially address health issues. Participants offered suggestions they believed could help decrease the risk of health-inhibiting effects, such as adding access to resources to help individuals cope. Additionally, this research supports previously documented health-enhancing benefits linked to mindfulness and MAs but raised concerns over the health-inhibiting effects reported by participants.

Health-inhibiting effects associated with mindfulness have been documented in research on face-to-face practices, but there is little evidence investigating these effects in emerging technology, specifically apps. Highlighting these effects is especially important in the wake of the COVID-19 pandemic, as MAs are being downloaded more than ever before [19]. Thus, we will discuss the factors that contribute to health-inhibiting effects from these findings in order to optimize their translational value and to help develop the best practices for safety regulations for these apps.

### 4.1. Identifying Health-Inhibiting Effects

Participants who developed a dependency on the apps explained how they relied on them to cope with mental distress (i.e., emotions) and to attempt to treat physical health issues (i.e., sleep). Research on traditional in-person interventions shows that individuals can become dependent on mindfulness, including both the techniques and their facilitators (e.g., spiritual teachers, therapists, instructors [54,55,56]. The findings of this study expand previous research on dependency and reliance, suggesting that individuals can also become dependent on mindfulness delivered via mHealth interventions, particularly commercial MAs. This study also demonstrates the impact mHealth dependency has on individuals’ health, as participants reported being unable to control their emotions (increasing mental distress) or fall asleep (impacting physical health) without the app. Participants who reported developing dependency suggested that app developers could enforce a usage limit on the apps as a strategy to deter reliance.

When participating in activities on the app, participants became hyper focused on the idea of “doing it right” (i.e., meditation). The resulting performance uncertainty was accompanied by feelings of frustration, anxiety, and disappointment, which were perpetuated by the lack of feedback from apps in comparison to face-to-face mindfulness interventions. This finding echoes previous research on MAs where users have reported feelings of uncertainty related to their performance. Clarke and Draper’s [28] study of university students using Calm found that participants were unmotivated to use the app “because they didn’t [don’t] know if [they’re] doing it right,” attributing the lack of motivation to “participants’ low self-efficacy” (p. 6).

Participants at times described how their use of the app backfired, exacerbating mental distress such as anxiety and stress and bringing on unwanted and intrusive thoughts. Although this has been documented in studies observing formal meditation practice [55,57,58], our results show that this increase in mental distress could be tied to the absence of feedback, which is unique to MAs. Adapting techniques used in traditional mindfulness interventions (i.e., face-to-face) to help decrease mental distress in MAs should be considered when developing best practices. Participants who experienced increased distress offered suggestions such as “chat communities” where users can communicate with one another about their experiences and seek advice, as well as suggesting resources to connect users with trained professionals.

Participants in this study also reported physical distress related to their MA use, including loss of appetite, difficulty sleeping, lethargy, and body discomfort. Numerous studies have documented impacted sleep quality as a result of traditional MBIs, including multiple case reports [59,60,61] and mixed-methods research [62,63]. Additionally, research has linked MBPs to lack of appetite [55,59,64], lethargy [64], and body discomfort [65,66]. Despite the significant body of research focused on this theme, apps have a limited capability of addressing physical ailments (i.e., no in-person diagnosis). App development teams should consider adding a general advisory statement that these products are not intended to replace medical treatments for pre-existing conditions or ailments and should not be used as such.

### 4.2. A Need for Guidance: Providing Users with Support

The experiences reported by participants when describing performance uncertainty and backfiring share a common thread: lack of support. In the Buddhist tradition, mindfulness is practiced under the supervision of teachers (and personal practice) [67]. This is also true for MBSR programs delivered face-to-face [5,68,69]. Research that has focused on challenges and difficulties associated with meditation have pointed out the importance of the teacher in the meditation process [57,58,67].

Neary and Schueller [70] refer to MAs that encourage practice without the guidance of a clinician or professional as “unsupported apps.” The potential risks associated with unsupported apps, especially for vulnerable populations such as EAs, are of major concern. As participants in this study suggested, developers should strive to incorporate resources that allow users to receive support from a trained professional. This can be as simple as providing the phone numbers of national help hotlines or as complex as building a network of licensed professionals providing 24/7 support for users. Applications such as Calm have incorporated a user check-in feature, as suggested by EAs in this study, that allows users to record their mood after meditating [71], which is an important step in providing individuals the opportunity to keep track of their feelings.

### 4.3. A Need for Education: Enhancing Users’ Awareness of the Risks

App users may be unaware that mindfulness practice can be associated with health-inhibiting effects such as mental distress or physical ailments. Mindfulness practices call on individuals to actively become aware of the present moment, observing their thoughts and sensations, which can sometimes induce fear, anxiety, negative thinking, and stress, among others [72]. Previous research examining the experiences of practitioners of mindfulness have shown that these sensations and “distress” are a normal part of the meditation process [58,65]. However, researchers have pointed out that practitioners are usually made aware of this through their teachers or reading texts [57,58,65].

In MAs, information educating users about potential health-inhibiting effects is lacking. Participants in our study recommended that apps highlight the pros and cons of mindfulness, including both the potential health benefits and health-inhibiting effects. Participants felt that providing this information would allow users to increase their awareness, knowledge, and even normalize negative experiences. They explicitly suggested that such information should be easily accessible and not buried in the app’s Terms and Conditions.

### 4.4. Limitations

While other studies have been conducted on college students’ experiences using MAs, to the best of the researchers’ knowledge, this is the first report on EAs not restricted to college students. Although, it should be noted that the mean age for the study was 23, and a majority of participants reported being college students. This may result in a somewhat myopic view being represented in our findings. However, this should not take away from the experiences reported by these participants, as they are relative to their current environment and should be viewed as such. Additionally, the qualitative design of this study was appropriate as a first step to explore this phenomenon but supplementing this study with quantitative data could help illuminate health-inhibiting effects of Mas in larger quantity, including other demographics (e.g., age, education, socio-economic status) and clinical populations (e.g., diagnosed with anxiety or depression).

## 5. Conclusions and Future Research

Research on MAs has typically focused on associated health-related benefits and functionality. This article is the first to identify both health-enhancing and health-inhibiting effects. It also addresses these effects on a vulnerable population of MA users. Findings show that individuals using MAs may encounter the same health-inhibiting effects found in traditional mindfulness approaches. Collectively, the results can inform several “best practices” for MA developers to reduce users’ risk of these health-inhibiting effects. We advise app developers to incorporate the following practices:

1. Integrate professional support through the app (e.g., licensed professionals, resources for National Hotlines) that provides therapeutic support as well as feedback on mindfulness practices enacted with the app.
2. Include education about both potential health benefits and health-inhibiting effects associated with the practice of mindfulness, and ensure this information is accessible and available prior to using the app.
3. Screen users during the app setup process to identify novices and users with pre-existing mental or physical health conditions to tailor the app to reduce risks of health-inhibiting effects (e.g., limiting usage, providing more introduction to the practice of mindfulness).

Future studies could examine the content of apps to identify whether apps are addressing health risks, and subsequent studies could test these “best practices” to ascertain whether the guidelines reduce the occurrence of health-inhibiting effects. It is imperative that researchers and practitioners attempt to strategically communicate potential health-inhibiting effects to app users—not to deter them from engaging with the app—but to promote awareness, reduce risks, and promote understanding about such experiences, which are not uncommon in mindfulness-based practices and interventions. Lastly, researchers should examine if app users’ expectations can inform health outcomes using quantitative methods such as surveys to understand if underlying correlations exist.

## Figures and Tables

**Table 1:** Participant demographics.

Gender	
Male	9 (40.9%)
Female	13 (59.1%)
Age	
Minimum	20 (min)
Maximum	29 (max)
Mean/standard deviation	23.73 (*M*), 3.07 (*SD*)
Race/ethnicity	
White	7 (31.8%)
Asian	6 (27.3%)
African America	4 (18.2%)
Hispanic	2 (9.1%)
Latinx	1 (4.5%)
American Indian or Alaskan Native	1 (4.5%)
Biracial	1 (4.5%)

**Table 2:** Motivation and reasons.

	Particpants (%)
Communication sources	18 (81.8%)
Personal recommendation	5 (22.7%)
Professional recommendation	4 (18.2%)
Organic searching	5 (22.7%)
Advertising	4 (18.2%)
Existing access	4 (18.2%)
School	2 (9.1%)
Work	2 (9.1%)
Usage *	
Easy access/convenience	13 (52%)
Health issues mentioned later	5 (22.7%)
Health issues **	7 (28%)
Anxiety/stress	3
Post-traumatic stress symptoms	1
Sleep	3
No reason	5 (20%)

* Participants provided more than one response; ** subgroups do not receive a percentage.
